# Mental Health Monitoring in Esports Athletes Using an mHealth App: Requirements Analysis and Usability Study

**DOI:** 10.2196/90140

**Published:** 2026-09-30

**Authors:** Leona Stolberg, Daniel Hieber, Peter Kuhn, Michael Hebel, Johannes Schobel

**Affiliations:** 1 DigiHealth Institute, Neu-Ulm University of Applied Sciences, Wileystraße 1, Neu-Ulm, Bavaria, 89231, Germany, 49 731 9762 ext 1631; 2 Institute of Medical Data Science, University Hospital Würzburg, Würzburg, Bavaria, Germany; 3 Institute of Neuropathology, Ulm University Hospital, Ulm, Baden-Wuerttemberg, Germany; 4 HNU Game Institute, Neu-Ulm University of Applied Sciences, Neu-Ulm, Bavaria, Germany

**Keywords:** mental health, mobile health, mHealth, usability evaluation, requirements analysis, esports, gamification, user-centered design

## Abstract

**Background:**

Esports athletes face substantial psychological stressors comparable to those experienced by traditional athletes, yet mental health tools tailored to their specific needs remain scarce.

**Objective:**

This study addresses this critical gap by introducing MindAthlete, a prototype for a user-centered mobile health (mHealth) app intended to enhance mental health monitoring and support among esports athletes.

**Methods:**

A 3-stage development process was used over 5 months. First, structured qualitative interviews were conducted online via Google Meet with 7 experienced (sports) psychologists recruited through snowball sampling within professional esports networks. The interviews explored current mental health monitoring practices and key feature requirements for MindAthlete. Data were analyzed using Mayring’s approach to qualitative content analysis. Subsequently, a 32-screen high-fidelity prototype was developed in Figma based on the identified requirements. In the final stage, usability testing was conducted with 25 semiprofessional and professional esports athletes recruited via convenience and snowball sampling. Participants interacted with the Figma prototype and completed an online questionnaire administered via LimeSurvey, which included demographic items, the validated 10-item System Usability Scale (SUS) rated on a 5-point Likert scale, and open-ended questions on navigation, aesthetics, and overall functionality. Inferential analyses included Mann-Whitney *U* tests, Kruskal-Wallis tests, and Spearman correlations. Open-ended responses were analyzed using Mayring’s content analysis.

**Results:**

Expert interviews identified 8 core features for mental health monitoring in esports, clustered around 3 themes: continuous self-monitoring, structured assessment, and intervention and insight. Athlete usability testing yielded a median SUS score of 77.5 (IQR 67.5-87.5), classifying usability as “good” and exceeding a benchmark median of 68.3 derived from comparable mHealth apps. No significant differences were found by sex, age, years of professional experience, or prior mHealth usage. Qualitative feedback highlighted strengths including intuitive navigation and appealing aesthetics, alongside areas for improvement such as shorter animations and clearer initial onboarding. However, findings must be interpreted in light of the sample’s demographic homogeneity, which limits generalizability to the broader esports population.

**Conclusions:**

This research provides a stakeholder-driven, empirically validated blueprint for esports-specific mental health monitoring via mHealth. Future work will focus on technical implementation, integration of wearable sensor data, and broader evaluations with larger, more diverse samples to confirm generalizability and enhance usability.

## Introduction

Esports is defined as playing video games regulated through ranking systems and official leagues via electronic devices, encompassing a spectrum from amateur players to elite professionals [1]. While esports participation broadly covers various levels, this work focuses specifically on competitive esports athletes operating at semiprofessional and professional tiers. As participation and competitiveness have grown, understanding the health profile of esports players has become a central focus. Recent systematic and scoping reviews indicate that esports participants do not inherently exhibit poorer baseline mental health than nonparticipants [2], although high-volume participation is associated with specific physical, lifestyle, and psychological vulnerabilities [3,4]. Crucially, psychological states and health risks differ across competitive tiers. Nonprofessional and amateur players primarily face lifestyle-related risks, such as sedentary behavior, elevated body fat, and gaming disorder tendencies [2]. In contrast, semiprofessional and professional athletes, who operate under structured team environments, contractual expectations, and tight competition schedules, face intense domain-specific stressors, including performance expectations, team conflicts, and demanding tournament schedules [4-7], which predict a high prevalence of mental health challenges [8,9]. Among competitive players, these multidimensional demands frequently result in elevated stress, anxiety, depression, burnout, sleep disturbances, and night-eating tendencies [10-13]. These conditions highlight an ethical imperative to address mental health concerns proactively in esports, as untreated issues can compromise immediate performance and long-term psychological well-being [7,14]. However, the esports industry still lacks sufficient mental health support systems [15]. While players report positive attitudes toward psychological support [16], hiring dedicated staff is costly (especially for smaller organizations), and continuous manual monitoring is time-consuming. Mobile health (mHealth) apps may offer a scalable alternative to streamline screening and ongoing monitoring [17-19].

Although mHealth tools have demonstrated efficacy in symptom tracking for broad clinical conditions such as stress, depression, and posttraumatic stress disorder [20-23], they target general psychopathology rather than performance-related distress and contextual triggers unique to competitive athletes. Furthermore, such generic tools frequently suffer from poor usability and low long-term engagement [24,25]. In sports specifically, mHealth apps have demonstrated feasibility for mood tracking, automated risk detection, and psychological skills training, though tool quality and evidence bases vary widely [17,26]. Blended models combining in-person and digital components have been proposed to enhance athlete accessibility and real-time clinical decision-making [27,28]. However, these sport-specific tools are typically embedded within colocated, traditional training environments, and their design assumptions around physical load, coaching structures, and recovery processes often do not align with the screen-bound workflows of esports team structures, limiting their transferability. The esports ecosystem itself has seen a rise in performance-enhancement tools lacking scientific validation [1], and players may also engage with generic mindfulness apps or game-embedded focus aids. However, these tools are designed to support general relaxation or productivity rather than to screen for or monitor mental health risk, offer no structured pathway to practitioner oversight, and do not capture the specific performance, organizational, and social stressors that distinguish this population [5,7], limiting their value as tools for proactive mental health support.

Given these limitations, we developed MindAthlete—a novel, noncommercial mHealth prototype designed specifically for mental health screening and monitoring in esports athletes. It is shaped through direct consultation with practicing esports psychologists and esports players, ensuring that each design decision reflects real-world clinical needs rather than assumed requirements.

To guide this development process, 2 research questions were formulated:

Research question 1: What features are essential for mental health monitoring in esports athletes according to (sports) psychologists?

Research question 2: How do esports athletes rate the usability of the prototype, and what do they identify as strengths, areas for improvement, and additional feature needs?

## Methods

### Study Design

In this study, we followed a 3-stage development process (Figure 1). This process was guided by the design thinking methodology, which emphasizes iterative, user-centered development and encourages early stakeholder involvement to ensure the solution fits real-world needs [29]. While user-centered design typically involves end users at every stage, our approach used a phased stakeholder engagement strategy. Given that MindAthlete is designed for a dual-user ecosystem (athletes for data entry and specialized practitioners for clinical monitoring), we prioritized the “expert-user” during the initial define and ideate phases. This choice was driven by the clinical necessity to ensure that the app features were scientifically valid and actionable for practitioners, who serve as the primary stakeholders for data interpretation and intervention. The first stage involved systematically gathering functional and nonfunctional requirements through structured interviews with domain experts. In the second stage, we created a design prototype to visualize the user interface and intended workflow. In the final stage, we conducted usability testing with semiprofessional and professional esports athletes to empirically evaluate MindAthlete’s usability and collect targeted feedback for further refinement.

**Figure 1.**
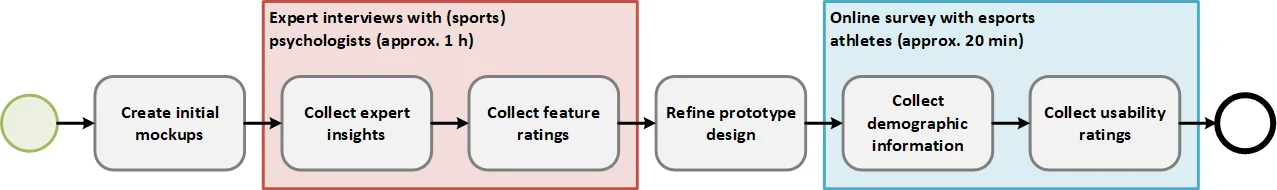
Overall study design. Red: expert interviews with (sports) psychologists; blue: online survey with esports athletes.

### Expert Interviews

The recruitment phase for the expert interviews followed a snowball sampling approach, as the population of practitioners specialized in esports psychology and mental health is extremely small globally. This scarcity is reflected in the broader industry reality, where dedicated sports psychology support remains absent across much of the professional landscape [15]. Leveraging initial contacts within a professional network via email, additional suitable experts were identified through recommendations, ensuring that participants had highly relevant and specialized expertise. Snowball sampling was particularly appropriate given the niche nature of the esports psychology field. Inclusion criteria required participants to hold an academic degree in psychology or sports psychology and to have at least 3 years of professional experience. Additionally, eligible participants had to be actively involved in the esports industry as practitioners, either working with athletes competing in higher divisions of the European Regional Leagues or providing guidance to professional esports players in international top-tier competitions. Finally, participants had to be proficient in German or English. These criteria were selected to ensure that only experts with a solid academic background and direct practical experience in supporting esports athletes’ mental health contributed to the study.

After the experts gave their informed consent, an online meeting was scheduled. The interviews followed a predefined interview guide to ensure a standardized procedure (Multimedia Appendix 1) and were conducted by the first author (LS), who possessed domain expertise in esports player performance and formal training in qualitative research methodologies. At the beginning of the interview, experts were asked about the necessity and current practices of mental health monitoring in esports. Then, they were introduced to mHealth apps and asked about the required features for MindAthlete. Eight potential features were preselected based on a literature review of mHealth apps for mental health and the evaluation of leading apps in this domain. This helped to identify core functionalities commonly recommended or implemented for psychological self-monitoring. These features were presented to the experts via screen sharing and explained in detail. Experts were then invited to provide feedback regarding the potential benefit of these features. Additionally, they could suggest additional features, which were then verbally refined in close collaboration. Experts were then asked to rank both the preselected and their own suggested features based on perceived importance and usefulness. While all experts ranked the 8 preselected features, the total number of ranked items varied because experts could also rank their own suggested features. However, the scale always remained fixed at 8 points for the highest-ranked item down to 1 point for the eighth. The 8 highest-ranked features across all interviews were selected for implementation. A point-based system was introduced, assigning 8 points to the highest-ranked feature, 7 to the second-ranked feature, and continuing in this manner until the eighth-ranked feature, which received 1 point. In cases of equal point totals, features were ranked by evaluating their specificity to esports mental health vs broader health contexts and prioritizing the feature with more immediate relevance to professional esports. Finally, experts were asked about their preferences regarding the user interface, platform accessibility, and data privacy concerns.

All interviews were recorded and automatically transcribed using the aTrain software [30] and manually checked for accuracy. Data analysis involved both deductive and inductive content analysis, following Mayring’s approach [31]. Two researchers were involved in the qualitative analysis. The first author (LS) conducted the initial coding. Deductive categories were derived from the interview guide and covered the perceived importance of mental health tracking, past and present monitoring methods, their sufficiency, comprehensive metrics for mental health assessment, feedback on each of the 8 literature-based candidate features, further expert-suggested features, interface preferences including colors and platform, data security concerns, feature ratings, and reasons for those ratings. The feature-related deductive categories were each coded along two dimensions: expert opinions on the feature and specific suggestions or requirements for its implementation. To capture unanticipated themes, inductive categories were derived directly from the transcripts following Mayring’s inductive category formation procedure. The criterion of selection was defined to include any statement addressing contextual factors, organizational conditions, or operational requirements not covered by the predefined feature matrix. Text segments meeting this criterion were open-coded, grouped by thematic similarity through iterative reading, and subsumed into broader categories (cooperation with support staff, the perceived purpose of the application, user engagement strategies, and challenges in application adoption). A senior supervisor (JS) reviewed the full code structure to ensure consistency and resolve discrepancies, providing feedback on category definitions and relevance (Multimedia Appendix 1). Following the coding process, categories related to feature ratings and expert rationale were used to calculate the point-based feature ranking system, while interface and contextual categories informed nonfunctional design decisions for the prototype.

### Design Prototype Development

The insights and user requirements identified through expert interviews were systematically aggregated, evaluated for coherence, and directly integrated into a high-fidelity design prototype using the prototyping software Figma [32]. A total of 32 screens were designed, representing the primary user flow from onboarding to feature interaction, including feedback elements and animations. The 8 highest-ranked features, as determined by experts, were implemented. Decisions to include, modify, or exclude certain feedback were guided by their alignment with expert consensus and evidence, and their coherence with established best practices identified in the literature. Additional feedback was retained if (1) the suggestion was voiced by at least 4 of the 7 experts, a threshold chosen to reflect clear majority consensus, or (2) the expert provided a concrete scientific or clinical rationale. Feedback that did not meet these criteria was archived for future iterations.

Low-fidelity mockups were iteratively refined into a high-fidelity prototype by the main author (LS) and the supervisor (JS), directly informed by the synthesis of the expert interviews. Choosing a high-fidelity prototype at this stage was justified by the need to closely simulate real user interactions, enabling an accurate assessment of usability. All screens and interaction flows were designed following user-centered design principles, drawing on Nielsen’s usability heuristics [33], Norman’s design principles [34], and empirical guidelines from health usability research [35], to ensure consistent layouts, clear navigation, and immediate feedback.

Applying these principles, the resulting prototype comprises the key features depicted in Figure 2. The Home Screen (Figure 2A) provides an overview of sessions, daily tips, and gamification status, with a sidebar for navigation. The Mood Tracker (Figure 2B) uses a slider and context tags to log current or daily mood. The Dashboard (Figure 2C) presents interactive graphs that users can filter by year, month, or week. Sport psychological questionnaires (Figure 2D) also include a weekly check-in for lifestyle data. The selection of specific validated instruments is ongoing and will be finalized in the next development phase. Instruments of interest are the World Health Organization-Five Well-Being Index (WHO-5 [well-being]), Generalized Anxiety Disorder-7 (GAD-7 [anxiety]), Patient Health Questionnaire-9 (PHQ-9 [depression]), Perceived Stress Scale (PSS [stress]), or Athlete Psychological Strain Questionnaire (APSQ [athlete-specific psychological strain]). Grounded in self-determination theory [36], gamification mechanics were incorporated to enhance engagement: each completed survey or tracked entry triggers a sky lantern animation (Figure 2E) that floats upward to symbolize progress and award points, ranks, and trophies, directly mirroring the reward systems native to competitive esports environments. This prototype formed the basis for subsequent usability testing.

**Figure 2.**
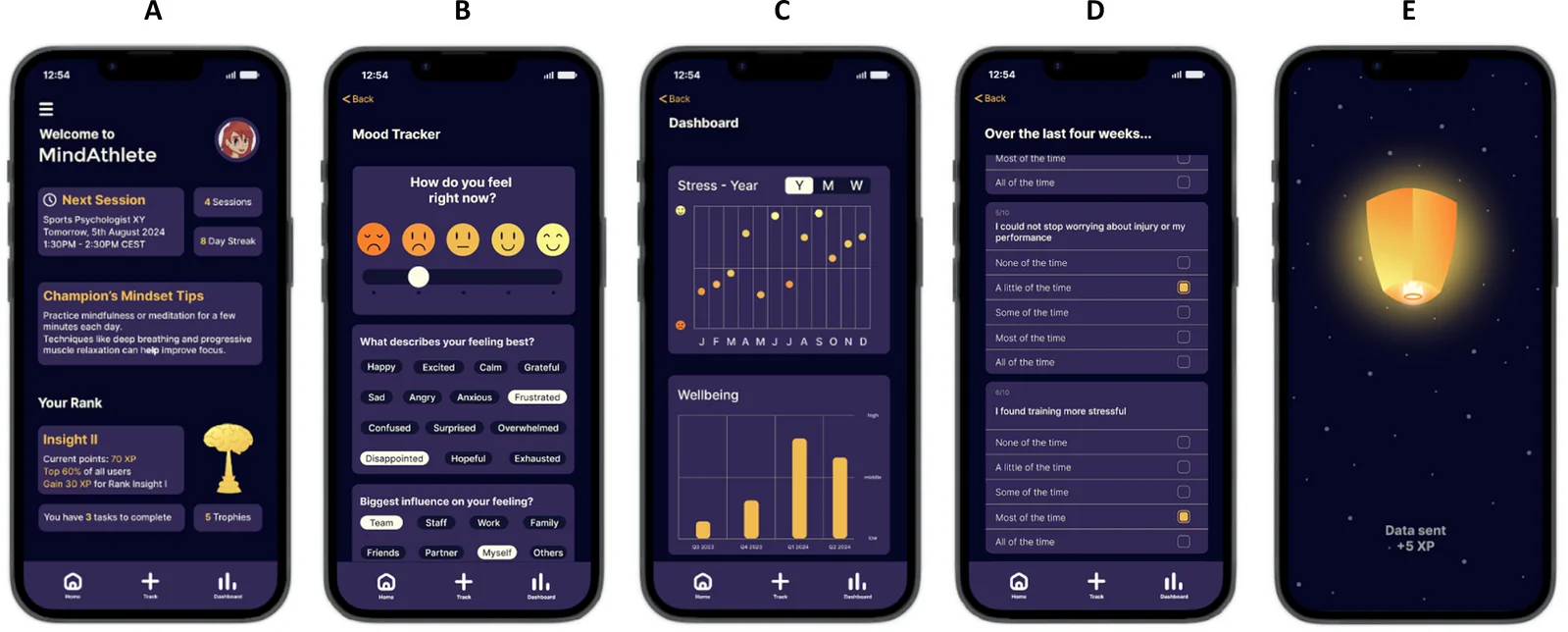
Screenshots of the Figma prototype. (A) Home Screen displaying the next session with the (sports) psychologist, daily tips, and gamification elements. (B) Mood Tracker allowing players to select their mood and add descriptive words. (C) Dashboard presenting results from the mood and stress trackers as well as questionnaires. (D) Example of a sport psychological questionnaire. (E) Sky Lantern animation representing the gamification theme and the metaphorical sense of letting go.

### Athlete Usability Testing

Participants were recruited through a combination of convenience and snowball sampling. Leveraging personal and professional contacts within the esports industry, initial invitations were distributed directly to esports athletes, teams, and affiliated organizations. Additional invitations were sent via professional esports networks to maximize outreach across different teams and regions using email and Discord, a primary communication platform within esports communities. Inclusion criteria required that participants be at least 18 years old and proficient in English, and actively competing in esports at a semiprofessional or professional level. Specifically, professional status was defined as athletes competing in top-tier continental leagues under full-time contracts, while semiprofessional status included athletes in regulated national or regional leagues (eg, Division 1 or 2). The participants in the latter category also operated under professional conditions, characterized by fixed salaries, official team affiliations, and commitment to training and competition. All participants provided their informed consent before participating.

Participants were invited to test the MindAthlete design prototype and complete an online questionnaire administered through LimeSurvey [37]. Usability was assessed using the validated 10-item SUS questionnaire, rated on a 5-point Likert scale [38]. The SUS was chosen due to its proven reliability and widespread acceptance in usability research. A target sample size of 20 to 30 participants was deemed sufficient based on recommendations for usability testing with the SUS [39]. Additionally, open-ended questions were included to solicit detailed feedback on navigation, aesthetics, and overall functionality of the prototype. The questionnaire also collected demographic and contextual information, including age, sex, nationality, esports experience (total and paid years), primary income status, and prior mHealth usage. All items were mandatory for completion, and the athletes were able to change their answers through a back button.

Data analysis consisted of three steps. First, descriptive statistics were computed to summarize the data. To determine the appropriate measures of central tendency, normality was assessed using the Shapiro-Wilk test, and outliers were examined using the IQR method. Second, inferential analyses were conducted to examine prespecified group differences and relationships. Specifically, SUS scores were compared across sex groups (female, male, and other) and age groups (18-21, 22-25, 26-29, and 30-33 y) using the Mann-Whitney *U* test and Kruskal-Wallis test, respectively. Associations between SUS scores and continuous variables (years of professional experience and paid years of experience) were assessed using Spearman correlations. Additionally, group comparisons were performed for main income status (esports as primary income: yes/no), prior mHealth usage (yes/no), and nationality. A Bonferroni correction was applied to control the family-wise error rate across all inferential tests. Each obtained *P* value was multiplied by the total number of comparisons performed (*k*=7) and compared against the nominal significance threshold of α=0.05. Finally, open-ended responses were analyzed qualitatively using Mayring’s content analysis approach [31] by the primary coder (LS) and reviewed by (JS), following the 2-tiered deductive-inductive procedure described previously. Top-level deductive domains were anchored directly to the 3 open-ended survey prompts: positive feedback, areas for improvement, and suggestions for additional features. Within each deductive domain, inductive subcategories were derived directly from the response text through line-by-line coding and iterative thematic grouping. For positive feedback, inductive analysis yielded 3 categories: perceived learnability and low cognitive load, aesthetic acceptance, and perceived safety through help-seeking visibility. Areas for improvement produced 5 categories: gamification fatigue, onboarding deficit, navigation ambiguity, information density, and individual usability concerns. Feature suggestions yielded 4 categories: behavioral prompting and habit formation, intelligent clinical integration, privacy-controlled communication, and holistic well-being expansion.

### Researcher Characteristics and Reflexivity

The research team possessed an interdisciplinary background combining expertise in game development, esports, and health informatics. The first author previously worked as a project manager within the esports industry. This prior insider knowledge aided in the contextual interpretation of data across both studies. The second author, serving as the supervisor, is an experienced researcher in digital health who reviewed all coding decisions to mitigate potential bias. These characteristics were considered throughout the research process, particularly during data analysis, where iterative discussions between both researchers aimed to balance insider familiarity with critical distance.

### Ethical Considerations

All participants were asked to give their informed consent after reading information about the purpose of the project, the investigators, the length of time, data protection, and the questionnaire domains. To safeguard participant information, all data were treated confidentially. Expert interview recordings were pseudonymized immediately after transcription, with participants assigned neutral identifiers and the original recordings subsequently deleted. Athlete questionnaire data were collected anonymously, with no personally identifiable information retained. Ethical aspects were reviewed and approved by the Joint Ethics Committee of the Universities of Applied Sciences of Bavaria (GEHBa) in accordance with current scientific best-practice guidelines (GEHBa-202403-V-170).

## Results

The following section presents the results of both studies, highlighting key findings and outcomes relevant to the research questions.

### Expert Interview Results

Structured qualitative interviews were conducted with 7 (sports) psychologists via Google Meet (Table 1). The participants represented 5 different nationalities and included sports psychologists (4/7, 57%) and general psychologists (3/7, 43%). The aim was to explore their perspectives on mental health monitoring in esports and to identify key requirements for MindAthlete.

**Table 1.** Information on interview partners and interview details.

Expert ID	Sex	Degree	Language	Duration, min
Expert 1	Female	Sports Psychology	German	108
Expert 2	Male	Sports Psychology	German	100
Expert 3	Female	Psychology	German	56
Expert 4	Female	Sports Psychology	English	95
Expert 5	Female	Psychology	German	50
Expert 6	Female	Psychology	German	81
Expert 7	Male	Sports Psychology	German	53

### Current Mental Health Monitoring Practices and Gaps

Experts affirmed the need for systematic monitoring given athletes’ vulnerability to mental health issues (perceived importance of mental health tracking). Current practice, relying on questionnaires, passive observation, and informal check-ins (past and present monitoring methods), is limited by subjectivity, declining engagement, and uncertain self-report honesty, as Expert 7 noted: “I’m just a bit dependent on it: do they tell me the truth?” (sufficiency of these methods). Comprehensive monitoring should integrate psychological, behavioral, emotional, and physical dimensions, with mood as an early indicator and an esports-adapted context (comprehensive metrics for mental health assessment). This is reflected in the prototype’s combined use of validated assessment tools, lifestyle-focused context input, and behavioral self-report tasks that pair quantifiable ratings with contextual, esports-specific detail, directly addressing experts’ concern that passive observation alone relies too heavily on subjective interpretation, while still capturing an esports-adapted picture that generic clinical tools do not offer.

### Feature Requirements

Experts evaluated 8 candidate features (feedback on literature-based candidate features), most strongly endorsing the mood and stress trackers and a dashboard providing trend views for both athletes and practitioners, as Expert 3 put it: “You need a dashboard where I can glance at trends, spreadsheets simply don't cut it.” Sport psychological questionnaires and mental health risk screening, both grounded in validated measures, were also well-supported. Stress relief techniques and help-seeking options were valued as around-the-clock resources, given esports’ irregular schedules. Experts also proposed features beyond the preselected set (expert-suggested features), most notably a weekly check-in capturing lifestyle contexts, such as sleep, social contacts, and fitness, alongside push notifications, journaling, goal-setting, and a buddy system, of which only the weekly check-in ranked among the top 8. The ratings (feature ratings and reasons for ratings) converged strongly across psychologists and sports psychologists, with data collection features rated highest, as Expert 4 summarized: “All the trackers are the most important bit for me...But what is the point of having trackers if you do not have a dashboard?” This convergence highlights a shared need to turn raw data into actionable clinical insight, not merely to collect it. The 8 highest-ranked features (Table 2) directly informed the final feature set, clustering into continuous self-monitoring (mood tracker, stress tracker, and weekly check-in), structured assessment (sport psychological questionnaires and mental health risk screening), and intervention and insight (stress relief techniques, help-seeking options, and dashboard), reflecting a balance between mental health monitoring and on-demand support. Help-seeking options were prioritized over messages in a tie-break, as there are other already existing channels. Psychoeducation, while supported, raised feasibility concerns and did not rank in the final 8.

**Table 2.** Top 8 features for MindAthlete and their total points.

Feature	Points
Mood tracker	48
Stress tracker	43
Dashboard	27
Sport psychological questionnaires	21
Stress relief techniques	20
Mental health risk screening	19
Weekly check-in	13
Help-seeking options	11

### Design and Implementation Considerations

Two deductive categories addressed practical design requirements. Experts favored nonclinical, gaming-familiar aesthetics, including a dark mode, a gaming-inspired color scheme, and consistently preferred desktop access for practitioners’ data review alongside mobile access for athletes (interface preferences including colors and platform). Both the dark mode and color scheme were incorporated into the mobile prototype, and the platform preference directly shaped MindAthlete’s dual-platform design: a mobile app for athletes and a web-based practitioner dashboard planned for future development. Experts also emphasized data security given the sensitivity of mental health data, recommending anonymized identifiers, biometric or PIN-based access, and transparent communication about data use (data security concerns), as Expert 4 noted: “Kind of considering the ethics of this, like where is the information going? Who has access to information?” These measures have not yet been implemented, having been deprioritized in favor of the top 8 core features and initial engagement mechanics.

### Broader Application Context

Experts envisioned MindAthlete as a support ecosystem that reduces practitioners’ administrative burden through preanalyzed data (purpose of the application), as Expert 1 put it: “I should not be looking at the data myself, it needs to land on my desk already analyzed.” They also anticipated adoption barriers, including practitioners’ limited review time, athletes’ limited emotional literacy and tendency toward socially desirable responding, and a self-reinforcing risk of declining engagement if athletes perceive no tangible benefit from their input (challenges in application adoption). A related category emphasized the need for shared understanding among all staff, including ways for nonspecialist staff such as coaches to contribute their own observations about an athlete (cooperation with staff). Finally, experts recommended sustaining engagement through positive reinforcement, gamification, and minimal interaction friction (user engagement strategies). These insights were reflected in several design decisions. To address athletes’ limited emotional literacy, the mood and stress trackers were designed with selectable response options, lowering the barrier to reporting. Experts’ engagement recommendations were directly operationalized through the prototype’s gamification features, confirming the relevance of the point-based mechanic and rank system. Their suggestions regarding staff cooperation have not yet been implemented. Together with the broader vision and adoption risks described above, these considerations extend beyond the current prototype’s scope and inform priorities for future development.

These expert insights directly address research question 1 by identifying and prioritizing the core features considered most relevant for mental health monitoring in esports.

### Athlete Usability Testing Results

A total of 134 esports athletes, identified as competitors in 1 of 5 different video games, were contacted for this study. This cohort primarily reflected the male-dominated demographic typical of the professional esports scene. After excluding 3 incomplete responses, the final sample comprised 25 participants. The sample was predominantly female (n=17, 68%) and heavily concentrated in the 22 to 25 age bracket (n=14, 56%). The cohort consisted almost entirely of League of Legends players (n=24, 96%), with an average professional experience of 4.8 (SD 3.2) years, and 17 of 25 (68%) relying on esports as their primary income source. Notably, 22 of 25 (88%) reported no prior experience with mHealth apps. Complete participant demographics are detailed in Table 3.

**Table 3.** Participant demographics (N=25).

Characteristic		Participants, n (%)
Age group (y)		
18‐21		2 (8)
22‐25		14 (56)
26‐29		8 (32)
30‐33		1 (4)
Sex		
Female		17 (68)
Male		7 (28)
Other		1 (4)
Nationality		
Germany		12 (48)
France		4 (16)
Great Britain		3 (12)
Spain		2 (8)
Other		4 (16)
Primary game		
League of Legends		24 (96)
EA Sports FC		1 (4)
Esports as main source of income		
Yes		17 (68)
No		8 (32)
Prior mHealth app usage		
Yes		3 (12)
No		22 (88)

The distribution of SUS scores significantly deviated from normality (Shapiro-Wilk *W* 0.915, N=25; *P*=.04), showing moderate left-skewness (skewness −0.81). Therefore, the median is reported as the primary measure of central tendency: median 77.5 (IQR 67.5‐87.5), range 42.5‐92.5, classifying as “good” usability according to established SUS benchmarks [40]. Analysis using the IQR method did not identify outliers (Table 4).

**Table 4.** Descriptive summary of System Usability Scale (SUS) scores overall and by sex and age.

Group	n (%)	Median (IQR); range
Total	25 (100)	77.5 (67.5‐87.5); 42.5‐92.5
Sex		
Female	17 (68)	75.0 (65.0‐82.5); 42.5‐92.5
Male	7 (28)	82.5 (73.8‐86.3); 47.5‐92.5
Other^a^	1 (4)	90.0^b^
Age (y)		
18-21^a^	2 (8)	80.0^b^
22‐25	14 (56)	73.8 (61.3‐79.4); 42.5‐90.0
26‐29	8 (32)	86.3 (79.4‐88.1); 47.5‐92.5
30-33^a^	1 (4)	82.5^b^

a SD is not reported for n<2. Data are left-skewed; the median (IQR) is reported as the primary measure.

b IQR and range are not reported for n<3.

Sex differences in SUS scores (female: n=17, 68%; male: n=7, 28%; other: n=1, 4%) were evaluated using the Mann-Whitney *U* test for female and male participants only. Females had a median SUS of 75.0 (IQR 65.0‐82.5), and males had 82.5 (IQR 73.8‐86.3). The Mann-Whitney test revealed no significant difference (*U*=74.0, *z*=−0.89, adjusted *P*>.99, *r*=0.19). Comparing age groups, the 22 to 25 (n=14, 56%) group showed a median SUS of 73.8 (IQR 61.3‐79.4), and the 26 to 29 (n=8, 32%) group had 86.3 (IQR 79.4‐88.1). Initially, the Mann-Whitney *U* test indicated a difference (*U*=26.5, *z*=−2.06, *P*=.047, *r*=0.43), but after Bonferroni correction, the adjusted *P*=.33, rendering the difference nonsignificant. Age groups with fewer than 5 participants (18‐21 y: n=2, 8%; 30‐33 y: n=1, 4%) were reported descriptively only. Spearman correlations between SUS scores and years of professional experience (ρ=0.14, adjusted *P*>.99) as well as paid experience (ρ=0.12, adjusted *P*>.99) were nonsignificant. A Mann-Whitney test comparing SUS by main income status (yes vs no) showed no significant difference (*U*=62.5, *z*=−0.29, adjusted *P*>.99, *r*=0.06). Similarly, prior mHealth usage yielded no significant difference (*U*=27.0, *z*=−0.47, adjusted *P*>.99, *r*=0.10). A Kruskal-Wallis test for nationality differences was nonsignificant (*H*[7]=6.29, adjusted *P*>.99), and small group sizes in some nationalities precluded further inferential analysis.

Table 5 summarizes the qualitative feedback provided by participants in response to the open-ended questions and is grouped into 3 domains: positive feedback, areas for improvement, and suggested features.

**Table 5.** Athlete qualitative feedback categories from open-ended responses (N=25).

Domain and category	Participants, n (%)	Description
Positive feedback		
Perceived learnability and low cognitive load	13 (52)	Interface experienced as immediately accessible without prior instruction
Aesthetic acceptance	7 (28)	Visual design perceived as engaging rather than clinical
Perceived safety through help-seeking visibility	5 (20)	Prominent emergency support options contributed to a sense of psychological safety
Areas for improvement		
Gamification fatigue	5 (20)	Lantern and trophy animations perceived as disruptive with repeated use
Onboarding deficit	3 (12)	Absence of a first-launch guide created uncertainty despite an intuitive interface
Navigation ambiguity	2 (8)	Bottom-menu icons lacked sufficient labeling
Information density	2 (8)	Home screen perceived as visually crowded
Individual usability concerns	3 (12)	Isolated issues, including a mobile scrolling bug, session-scheduling stress, and ranking-system confusion
Suggestions for additional features		
Behavioral prompting and habit formation	4 (16)	Reminders and alarms requested to sustain daily tracking behavior
Intelligent clinical integration	3 (12)	Desire for automated follow-up recommendations linking data to professional support
Privacy-controlled communication	2 (8)	Need for a private reflection space and a separate staff communication channel
Holistic well-being expansion^a^	1 (4)	Nature-sound relaxation, gamified daily-streak tracker, sleep and nutrition tracking, weekly summary dashboard

a Each suggestion was raised by a single participant and did not meet the threshold for a distinct category.

Positive feedback centered on ease of use, most prominently in the perceived learnability and low cognitive load category (13/25, 52%), reflecting athletes’ experience of the interface as immediately accessible without prior instruction. As P3 remarked, “The basic level of the UI made it feel very easy to understand and use when needing to enter information, making conveying what I felt into the application be done confidently.” Athletes also perceived the visual design as engaging rather than clinical and reported that prominent help-seeking options contributed to a sense of psychological safety. Together, these categories suggest that the design achieved both functional usability and the emotional accessibility necessary for engaging with sensitive self-monitoring content.

Critical feedback concentrated on motivational elements, initial orientation, and visual clarity rather than core functionality. The most frequently cited concern was gamification fatigue (5/25, 20%): while gamification elements were implemented to enhance engagement, the animations were perceived as disruptive with repeated use, as P1 noted, “the speed at which the lantern and trophy animations are played...feels like it could become annoying.” Onboarding and interface clarity issues were also raised. These findings suggest that MindAthlete’s core design foundations are sound, with targeted refinements needed in motivational calibration and onboarding.

Suggested features extended beyond aesthetic preferences toward a more integrated vision of the app. The most common request was for behavioral prompts such as reminders and alarms to sustain daily tracking (4/25, 16%), followed by a desire for automated follow-up recommendations that link daily data to professional support, reinforcing the dual-user ecosystem underlying the prototype’s design.

These findings provide a direct answer to research question 2, demonstrating that the prototype is perceived as user-friendly while also highlighting concrete areas for refinement and future development.

## Discussion

The following section discusses the key findings of this study, reflects on methodological limitations, and outlines implications for future research and development.

### Interpretation of Key Findings

Experts’ unanimous recognition of mental health tracking as a core professional responsibility reflects esports athletes’ high vulnerability to burnout and anxiety, as well as related stressors such as performance pressure and demanding schedules [5,6,10-12], with untreated issues compromising both immediate performance and long-term well-being [5,14]. This is also consistent with documented pathways from esports-specific stressors to mental ill health [8], including burnout [12] and night-eating [13]. Furthermore, experts’ emphasis on comprehensive mood tracking and dashboard visualization mirrors findings that early mood deterioration predicts severe episodes [41] and that continuous data visualization supports clinical decision-making [25,42], while future sensor integration enriches self-reported psychological data [43]. Feature ratings indicate an underlying rationale: partial deployment (eg, providing tracking features without an integrated dashboard) may limit their practical value. This suggests that MindAthlete’s features may be most useful when considered together, rather than as fully independent components. Inductive categories highlight engagement and organizational integration as central challenges. The fear of a self-reinforcing cycle in which absent feedback leads to indifferent athlete input mirrors persistent engagement drop-off documented across mHealth self-reporting tools more broadly [24,25], underscoring that closing the feedback loop is essential for sustained use. Similarly, organizational integration may be as important as technical functionality, consistent with evidence that workflow integration, institutional culture, and staff engagement are among the most prevalent determinants of mHealth adoption, often outweighing technological considerations [44].

MindAthlete’s usability score exceeded the benchmark median of 68.3 for mHealth apps, excluding physical activity applications [45], aligning with comparable athlete-facing tools ranging from 72.89 to 78.5 [46-48], despite being an unpolished prototype. Athletes’ qualitative feedback suggested that MindAthlete achieved emotional accessibility alongside functional usability, a distinction SUS scores alone cannot capture, particularly relevant given evidence that stigma and embarrassment limit engagement with mental health apps [49]. This is consistent with evidence that esports players welcome personalized, trustworthy support [16], suggesting MindAthlete may address these expectations. Furthermore, their requests for behavioral prompts and privacy controls reinforce health informatics research highlighting the necessity of trust and social scaffolding for long-term engagement [50].

Traditional sports mHealth tools often focus on physical workload, psychological skills training, or mood tracking, but are not designed for esports-specific stressors and rhythms [17,26], while generic mental health apps rely on nonspecialized screening and face persistent engagement challenges [23-25]. By grounding assessment categories, terminology, and gamification mechanics directly in esports culture, MindAthlete represents an initial step toward adapting mHealth principles to the specific performance context of esports.

### Potential Implications for Practice

These findings suggest several potential applications for MindAthlete beyond the current prototype stage, though each would require further development and validation. For organizations without dedicated psychological staff, the always-on trackers and screening features may offer accessible first-line support, and experts noted that a future practitioner dashboard could help nonspecialist staff recognize concerning trends. In organizations with existing psychological support, such a dashboard could complement rather than replace informal monitoring practices. More broadly, MindAthlete’s approach is consistent with blended models that combine digital self-monitoring with in-person or remote psychological support, which have been associated with improved continuity of care [27,28], though realizing this potential would require careful attention to data ownership and escalation protocols in future iterations.

### Limitations

Several limitations merit acknowledgment. First, relying primarily on experts during requirements gathering risked overlooking athlete-led innovations from lived experience. Experts were used exclusively at this stage because they could ground candidate features in established clinical and sport psychology practice, and we mitigated the resulting bias by validating and extending these features through athlete usability testing and open-ended qualitative feedback (research question 2). Only 7 experts were recruited, though strong thematic convergence suggests key requirements were captured despite the absence of full saturation. Snowball sampling may have introduced homogeneity among participating experts, and no formal intercoder reliability statistics were calculated, though a senior supervisor reviewed the coding structure to resolve discrepancies. Additionally, implementation constraints limited the prototype to 8 features, though experts could still propose novel features beyond this set to capture insights outside our literature-based preselection. Suggestions varied considerably across experts, but one novel feature was requested multiple times and ultimately incorporated. While this open approach increased data variability, it enriched the evaluation with previously unconsidered practitioner perspectives.

Regarding the usability testing, additional limitations exist that must be considered. Most notably, 68% of participants were female, a marked mismatch with the male-dominated professional esports industry. Although 134 predominantly male athletes were contacted, the final sample reflects strong self-selection toward female participation, consistent with evidence that men are less likely to engage in routine health-related help-seeking and survey participation [51,52]. Because engaging with a mental health application can itself be understood as help-seeking behavior, usability scores and open-ended feedback should be interpreted with caution. The sample was also highly homogeneous, concentrated in League of Legends (96%) and Germany (48%), reflecting the regional concentration of the European esports ecosystem and our recruitment network within it. Regional differences in working hours, training structures, and health-seeking norms—for instance, the considerably longer and less regulated training hours reported among professional esports athletes in China [53]—may limit the generalizability of usability perceptions to less structured competitive environments.

Relatedly, the cognitive and physiological demands of League of Legends as a Multiplayer Online Battle Arena (MOBA) title differ from those of other genres, with First-Person Shooter titles eliciting greater cardiovascular stress than MOBA titles [54], and MOBA gameplay emphasizing strategic planning and coordination over the reaction-time demands of First-Person Shooter and Real-Time Strategy titles [1]. These genre-specific profiles are relevant to the feature set, as stressors such as team communication breakdowns are structurally absent in individual titles such as fighting games or sim racing, and League of Legends’ team size may have provided athletes with more immediate social support, potentially reducing perceived need for MindAthlete.

Finally, only athletes aged 18 and older were included, and evaluations were based on a high-fidelity prototype rather than a final product. Motivation loss, notification fatigue, and long-term engagement could not be assessed, and positive usability scores may not reflect sustained real-world use. Cultural or prior experience factors may also have influenced the interpretation of SUS items.

### Conclusions

This study addressed the need for efficient mental health monitoring in esports by defining practitioner-driven requirements through expert interviews, translating them into an interactive prototype, and evaluating its usability with esports athletes. By grounding the prototype in esports-specific expert and athlete input, this study offers a practical foundation for future mental health tools tailored to the competitive esports context, rather than adapted from generic or traditional sports solutions. MindAthlete’s median SUS score of 77.5 demonstrates good usability for this niche audience. However, these findings must be interpreted in light of the sample’s demographic homogeneity. Future work should begin with the technical implementation of the mobile app and a web-based practitioner dashboard, the development of a validated crisis intervention protocol, and the establishment of robust data privacy and security mechanisms, developed together with (sports) psychologists, who are also being consulted on instrument selection and escalation management. Athlete feedback from this study should inform the next design iteration. Moreover, further studies should be conducted with larger sample sizes. Specifically, future research must deliberately target cross-cultural and longitudinal studies, focusing on non-European regions, other competitive game titles, and more male-dominated cohorts to explore whether usability and feature preferences differ, and to assess long-term engagement. Finally, integrating passive data collection via wearables could further enable objective monitoring and remote intervention, though this would require careful management of data overload, false positives, and privacy concerns.

## Supplementary material

10.2196/90140 Multimedia Appendix 1 Interview guideline and codebook.

## Abbreviations

APSQ: Athlete Psychological Strain Questionnaire
GAD-7: Generalized Anxiety Disorder-7
mHealth: mobile health
MOBA: Multiplayer Online Battle Arena
PHQ-9: Patient Health Questionnaire-9
PSS: Perceived Stress Scale
SUS: System Usability Scale
WHO-5: World Health Organization-Five Well-Being Index
