# Supplementary material for: Mental Health Monitoring in Esports Athletes Using an mHealth App: Requirements Analysis and Usability Study
Source: JMIR Hum Factors. 2026 Sep 30;13:e90140. doi: 10.2196/90140 (PMC13626294; doi:10.2196/90140)
Supplement: Supplementary file 1 [file humanfactors-v13-e90140-s001.docx]

**Appendices**

**Appendix 1 - Interview Guide**

**1.1 Introduction**

Topic: Requirements analysis and conception of an mHealth application for monitoring stress and mental health in esports athletes

• Audio Recording and Data Protection

• Project Description

• Definition Mental Health

**1.2 Block A - Current Situation**

Opening question: To what extent do you consider it necessary to systematically monitor the mental health and stress of your athletes?

***1.2.1 Scenario A - Not actively monitored at all:***

• Can you explain the reasons why you currently do not monitor the mental health and stress levels of athletes?

• How informed are you about the current stress and mental health of your athletes?

• How and to what extent would you want to monitor the mental health and stress of athletes?

• What data and metrics do you consider important for monitoring and assessing the mental state and stress of esports athletes?

***1.2.2 Scenario B - Actively monitored:***

• What insight do you have into the current stress and mental health of your athletes?

• Do you see opportunities for improvement or are there areas that you believe should be given greater consideration?

• What data and metrics do you consider important for monitoring and assessing the mental state and stress of esports athletes?

**1.3 Block B - Significance and Functions**

• Explanation of mHealth applications

• What specific functions or tools would you like to see in an application for monitoring stress and mental health of esports athletes?

**1.4 Block C - Ranking of the Features**

• Explanation of every prepared and suggested feature

• Introduction of functions ranking

• Confirm ranking + reason

**1.5 Block D - Additional Considerations**

• Do you have any preferences regarding the color scheme or layout of the user interface?

• Would you prefer using the application on a PC, on a phone or tablet, or on both?

• What requirements do you think should be considered regarding the privacy and security of the sport psychological data collected and stored in the software?

• Are there any other aspects that you consider important for this application?

**1.6 Summary of the Interview**

**Appendix 2 - Codebook**

# **2.1 Expert Interview Codebook**

Coding Approach: Mayring's qualitative content analysis. Deductive categories were derived a priori from the interview guide. Inductive categories emerged organically from the data during analysis. Initial coding was conducted by the first author, the full code structure was reviewed by a senior supervisor to ensure consistency and resolve discrepancies.

## **Deductive Categories**

**D1 – Importance of Mental Health Tracking**

**Definition:** Discussion on the significance of tracking mental health, including both perceived benefits and potential drawbacks.

**Coding Rule:** All statements that discuss the importance of mental health tracking, whether highlighting its benefits or acknowledging its limitations or lack of importance, are categorized here.

**Quote Example:** *"It's important, especially when we see how many people end their careers due to burnout and panic attacks."*

**D2 – Past and Present Methods for Monitoring Mental Health**

**Definition:** Description of past and current methods of mental health monitoring with esports players.

**Coding Rule:** All statements that describe specific methods, approaches, or practices for monitoring the mental health of esports players are categorized here. This includes both past and present practices.

**Quote Example:** *"I asked every player in person at least once a day, how are you?"*

**D3 – Sufficiency of Past and Present Methods**

**Definition:** Evaluation of whether current methods for mental health monitoring in esports are sufficient.

**Coding Rule:** All statements assessing the adequacy, effectiveness, or limitations of existing methods for monitoring mental health in esports players are categorized here. This includes both positive and negative evaluations.

**Quote Example:** *"I wouldn't think that I would notice everything with just observations, I don't think so."*

**D4 – Comprehensive Metrics for Assessing Mental Health**

**Definition:** Identification of key mental health metrics or dimensions relevant to esports players.

**Coding Rule:** All statements mentioning specific mental health metrics or dimensions that are important for monitoring the well-being of esports players are categorized here.

**Quote Example:** *"So I would ask about it on these levels: physically, mentally, but then also the behavioral level and also emotionally, [...]"*

**D5 – Feature: Questionnaires**

***Opinion about questionnaires feature:***

**Definition:** Personal opinions or subjective feedback on the questionnaires feature for mental health monitoring.

**Coding Rule:** All statements expressing opinions, whether positive or negative, about the use of questionnaires as a feature for monitoring mental health are categorized here.

**Quote Example:** *"It's very helpful and a very simple and intuitive way to collect data, I would say."*

***Suggestions and requirements for questionnaires feature:***

**Definition:** Examples, advice, suggestions, or essential requirements for improving the questionnaires feature.

**Coding Rule:** All statements where participants give specific examples, advice, suggestions, or essential requirements for the questionnaires feature.

**Quote Example:** *"The only thought that occurred to me was that you have to make sure that the questionnaires are freely accessible."*

**D6 – Feature: Risk for Mental Health Diseases**

***Opinion about risk for mental health diseases feature:***

**Definition:** Personal opinions or subjective feedback on the feature assessing risk for mental health diseases.

**Coding Rule:** All statements expressing opinions, whether positive or negative, about the use of risk assessment features for mental health are categorized here.

**Quote Example:** *"Yeah, I think this would be cool."*

***Suggestions and requirements for risk for mental health diseases feature:***

**Definition:** Examples, advice, suggestions, or essential requirements for improving the risk assessment feature.

**Coding Rule:** All statements where participants give specific examples, advice, suggestions, or essential requirements for the risk assessment feature.

**Quote Example:** *"I believe that if there is a suspicion in such a questionnaire or in a conversation with a sports psychologist that there may be an indication, then the next step would be to fill out a specific questionnaire."*

**D7 – Feature: Mood Tracker**

***Opinion about mood tracker feature:***

**Definition:** Personal opinions or subjective feedback on the mood tracker feature for mental health monitoring.

**Coding Rule:** All statements expressing opinions, whether positive or negative, about the use of mood trackers as a feature for monitoring mental health are categorized here.

**Quote Example:** *"So I think that's really nice and easy for them to use."*

***Suggestions and requirements for mood tracker feature:***

**Definition:** Examples, advice, suggestions, or essential requirements for improving the mood tracker feature.

**Coding Rule:** All statements where participants give specific examples, advice, suggestions, or essential requirements for the mood tracker feature.

**Quote Example:** *"So I think you have to think about how often this would be filled out?"*

**D8 – Feature: Stress Tracker**

***Opinion about stress tracker feature:***

**Definition:** Personal opinions or subjective feedback on the stress tracker feature for mental health monitoring.

**Coding Rule:** All statements expressing opinions, whether positive or negative, about the use of stress trackers as a feature for monitoring mental health are categorized here.

**Quote Example:** *"Yeah, I definitely think it's necessary."*

***Suggestions and requirements for stress tracker feature:***

**Definition:** Examples, advice, suggestions, or essential requirements for improving the stress tracker feature.

**Coding Rule:** All statements where participants give specific examples, advice, suggestions, or essential requirements for the stress tracker feature.

**Quote Example:** *"That's why it might be easier to do it with 3-4 questions."*

**D9 – Feature: Dashboard**

***Opinion about dashboard feature:***

**Definition:** Personal opinions or subjective feedback on the dashboard feature for mental health monitoring.

**Coding Rule:** All statements expressing opinions, whether positive or negative, about the use of dashboards as a feature for monitoring mental health are categorized here.

**Quote Example:** *"Dashboard with visualization is an absolute basic requirement for me, which I need when I have so many questionnaires and surveys."*

***Suggestions and requirements for dashboard feature:***

**Definition:** Examples, advice, suggestions, or essential requirements for improving the dashboard feature.

**Coding Rule:** All statements where participants give specific examples, advice, suggestions, or essential requirements for the dashboard feature.

**Quote Example:** *"That you have a performance coach profile and a psychologist profile."*

**D10 – Feature: Psychoeducation**

***Opinion about psychoeducation feature:***

**Definition:** Personal opinions or subjective feedback on the psychoeducation feature for mental health monitoring.

**Coding Rule:** All statements expressing opinions, whether positive or negative, about the use of psychoeducation as a feature for monitoring mental health are categorized here.

**Quote Example:** *"But I think there is some benefit to this idea of like psychoeducation and like extra stuff."*

***Suggestions and requirements for psychoeducation feature:***

**Definition:** Examples, advice, suggestions, or essential requirements for improving the psychoeducation feature.

**Coding Rule:** All statements where participants give specific examples, advice, suggestions, or essential requirements for the psychoeducation feature.

**Quote Example:** *"For the topics that interest you. And a search function."*

**D11 – Feature: Help-Seeking**

***Opinion about help-seeking feature:***

**Definition:** Personal opinions or subjective feedback on the help-seeking feature for mental health monitoring.

**Coding Rule:** All statements expressing opinions, whether positive or negative, about the use of help-seeking as a feature for monitoring mental health are categorized here.

**Quote Example:** *"Really good. Really important that it's right there. As easy as possible access to people who can help."*

***Suggestions and requirements for help-seeking feature:***

**Definition:** Examples, advice, suggestions, or essential requirements for improving the help-seeking feature.

**Coding Rule:** All statements where participants give specific examples, advice, suggestions, or essential requirements for the help-seeking feature.

**Quote Example:** *"It's like 3 a.m. and I'm asleep, it's pretty unlikely that I'm going to answer. Maybe there is something that can be added about hours of availability."*

**D12 – Feature: Tasks**

***Opinion about tasks feature:***

**Definition:** Personal opinions or subjective feedback on the tasks feature for mental health monitoring.

**Coding Rule:** All statements expressing opinions, whether positive or negative, about the use of tasks as a feature for monitoring mental health are categorized here.

**Quote Example:** *"Yes, I find it efficient.”*

***Suggestions and requirements for tasks feature:***

**Definition:** Examples, advice, suggestions, or essential requirements for improving the tasks feature.

**Coding Rule:** All statements where participants give specific examples, advice, suggestions, or essential requirements for the tasks feature.

**Quote Example:** *"I would, if then add a reminder to the tasks.“*

**D13 – Suggested Features**

**Definition:** Description of the features and characteristics proposed by interviewees for an mHealth application for mental health monitoring.

**Coding Rule:** All statements containing specific suggestions or recommendations for features and characteristics of an mHealth application are categorized here.

**Quote Example:** *"Mindfulness exercises, relaxation exercises, meditation, breathing exercises, everything."*

**D14 – Color**

**Definition:** Discussions about color schemes and their importance or impact on the mHealth application.

**Coding Rule:** All statements about the choice, significance, or impact of colors in the mHealth application are categorized here. This includes preferences, psychological impacts, and design considerations.

**Quote Example:** *"So the dark actually appeals to me, I think it's also kind to the eyes."*

**D15 – Platform**

**Definition:** Which platform the experts want to use the system on.

**Coding Rule:** All statements regarding which platforms the mHealth application should be available on, and any platform-specific considerations, are categorized here.

**Quote Example:** *"As a psychologist, I would rather use it on my PC than on my cell phone."*

**D16 – Data Security**

**Definition:** Concerns and requirements regarding data security and privacy for the mHealth application.

**Coding Rule:** All statements related to the importance of data security, privacy measures, and protocols for handling sensitive information are categorized here.

**Quote Example:** *"It's super important because sensitive data is also tracked."*

**D17 – Feature Ratings**

**Definition:** Numerical or ranked evaluations assigned to features during the ranking exercise.

**Coding Rule:** All statements containing explicit rankings, point assignments, or comparative evaluations of features during the structured ranking task are categorized here.

**Quote Example:** *"All the trackers are the most important bit for me. They provide a quick and easy way to get information from the players."*

**D18 – Reasons for Ratings**

**Definition:** Explanatory statements provided by experts to justify their feature rankings.

**Coding Rule:** All statements where experts explain the rationale behind their feature rankings are categorized here. Statements must relate directly to a previously expressed ranking.

**Quote Example:** *"But what is the point of having trackers if you do not have a dashboard?"*

## **Inductive Categories**

**I1 – Challenges in Application Adoption**

**Definition:** Anticipated barriers to real-world implementation, including practitioner workload, athlete-side resistance, and the self-reinforcing indifference cycle.

**Coding Rule:** All statements outlining specific challenges or obstacles faced in the process of mental health monitoring or implementing mHealth applications are categorized here. This includes technical, logistical, and psychological challenges.

**Quote Example:** *"They don't know exactly, do I feel anger, am I disappointed or something else. They can't quite put their finger on it."*

**I2 – Cooperation with Staff**

**Definition:** Desired level and type of cooperation between staff (including sports psychologists) and players, and the organizational conditions necessary for successful adoption.

**Coding Rule:** All statements expressing wishes or expectations for the type and extent of cooperation between esports players and staff are categorized here. This includes communication, support, and collaborative efforts.

**Quote Example:** *"So they say, okay, we have to do this now. That's crucial, a sense of purpose, make it clear to the athletes."*

**I3 – Purpose of the Application**

**Definition:** Desired purposes and goals of the mHealth application from the perspective of the experts.

**Coding Rule:** All statements expressing the intended purposes and goals that experts hope the mHealth application will achieve are categorized here.

**Quote Example:** *"I would have someone else analyze it for me anyway. I'm not supposed to look at the data myself, it has to end up in my office somehow."*

**I4 – User Engagement Strategies**

**Definition:** Approaches aimed at enhancing user interaction, participation, and satisfaction, according to the experts.

**Coding Rule:** All statements describing specific strategies, features, or design principles intended to improve user engagement and sustained usage are categorized here.

**Quote Example:** *"So if possible, break down barriers and increase engagement through gamification or something similar."*

# **2.2 Athlete Usability Testing Codebook**

Coding Approach: Mayring's qualitative content analysis applied to open-ended survey responses. Deductive categories were derived from the three open-ended question topics. Inductive subcategories emerged from the response content during analysis within each deductive category.

## **Deductive Category: Positive Feedback**

**A1 – Perceived Learnability and Low Cognitive Load**

**Definition:** Statements indicating that the interface was immediately accessible and required minimal instruction to begin using.

**Coding Rule:** All statements where participants express that the application was intuitive, easy to understand, or required no prior instruction are categorized here. Exclude statements about visual design preferences.

**Quote Example:** *"The basic level of the UI made it feel very easy to understand and use when needing to enter information, making conveying what I felt into the application be done confidently."*

**A2 – Aesthetic Acceptance**

**Definition:** Statements expressing that the visual design was perceived as engaging, appealing, or non-clinical.

**Coding Rule:** All statements where participants comment positively on the visual appearance, color scheme, or overall look and feel of the application are categorized here. Exclude statements about ease of use or navigation.

**Quote Example:** *"The colors and design was nice."*

**A3 – Perceived Safety through Help-Seeking Visibility**

**Definition:** Statements indicating that the prominent visibility of support options contributed to a sense of psychological safety or emotional comfort.

**Coding Rule:** All statements where participants express feeling safe, supported, or heard due to the accessibility of help-seeking features are categorized here.

**Quote Example:** *"Je me sens safe, ecouté et comprise. [translated: I feel safe, heard and understood.]"*

## **Deductive Category: Areas for Improvement**

**A4 – Gamification Fatigue**

**Definition:** Statements indicating that motivational animations or gamification elements were perceived as disruptive, excessive, or potentially annoying with repeated use.

**Coding Rule:** All statements where participants critique the frequency, speed, or intrusiveness of gamification elements are categorized here. Exclude general negative feedback about the visual design.

**Quote Example:** *"The speed at which the lantern and trophy animations are played ... feels like it could become annoying.)"*

**A5 – Onboarding Deficit**

**Definition:** Statements indicating that the absence of an initial orientation guide created uncertainty or confusion during first use.

**Coding Rule:** All statements requesting a tutorial, guided tour, or introductory walkthrough for first-time users are categorized here.

**Quote Example:** *"A tour of the app on first launch ... would be good."*

**A6 – Navigation Ambiguity**

**Definition:** Statements indicating confusion regarding interface navigation, particularly regarding menu icons or structural layout.

**Coding Rule:** All statements where participants express uncertainty about how to navigate between screens or interpret navigation elements are categorized here. Exclude comments about visual density.

**Quote Example:** *"when you open the menu down middle by pressing on 'track', there are 3 images that pop. those images aren't suggestive enough / intuitive as to what they represent.”*

**A7 – Information Density**

**Definition:** Statements indicating that certain screens were perceived as visually crowded or overwhelming.

**Coding Rule:** All statements where participants describe screens as containing too much information or feeling visually cluttered are categorized here. Exclude navigation-related confusion.

**Quote Example:** *"In my opinion the home interface may be a bit too crowded and there's too many information on the screen"*

**A8 – Individual Usability Concerns**

**Definition:** Isolated, actionable usability issues not captured by other categories, including technical bugs, scheduling concerns, and interface confusion.

**Coding Rule:** All statements describing specific technical bugs, interface errors, or singular usability issues that do not fit into the categories above are categorized here.

**Quote Example:** *"On the mobile version I couldn't scroll down easily to the bottom end of the page to save my questionnaire.”*

## **Deductive Category: Suggestions for Additional Features**

**A9 – Behavioral Prompting and Habit Formation**

**Definition:** Suggestions for external scaffolding mechanisms to support consistent self-monitoring behavior.

**Coding Rule:** All statements requesting reminders, alarms, or automated prompts to encourage regular app engagement are categorized here.

**Quote Example:** *"An alarm to remind you of things..."*

**A10 – Intelligent Clinical Integration**

**Definition:** Suggestions for automated decision support linking self-monitoring data to professional psychological resources or follow-up actions.

**Coding Rule:** All statements requesting automated recommendations, alerts, or clinical linkage based on tracked data are categorized here. Exclude general requests for reminders.

**Quote Example:** *"Des conseils liés aux réponses des différents questionnaires, par exemple si je souffre de dépression sévère: de la documentation dessus, des recommandations pour consulter un professionnel de santé, un accompagnement." [translated: Advice linked to questionnaire responses, for example if I suffer from severe depression: documentation on it, recommendations to consult a health professional, support.]”*

**A11 – Privacy-Controlled Communication**

**Definition:** Suggestions for features enabling private personal reflection alongside optional structured communication with support staff.

**Coding Rule:** All statements expressing a desire for control over data visibility or requesting a private space within the application are categorized here.

**Quote Example:** *"Private Area (also with Journaling Feature) and separated shared area for communicating with staff."*

**A12 – Holistic Wellbeing Expansion**

**Definition:** Suggestions for extending the application beyond mental health monitoring toward broader self-management features.

**Coding Rule:** All statements proposing features related to sleep, nutrition, physical activity, or general wellbeing tracking that go beyond the current feature set are categorized here.

**Quote Example:** *"Some sort of summary of weekly or monthly data collected - can also motivate to keep using the app."*
